# Construction and validation of a novel prognostic nomogram for predicting overall survival in lung adenocarcinoma patients with different patterns of metastasis

**DOI:** 10.1007/s00432-023-05288-8

**Published:** 2023-08-23

**Authors:** Ying Xiong, Feifei Gu, Jin Cui, Yuting Liu, Min Sun, Xinyue Gu, Luhui Zhong, Kai Zhang, Li Liu

**Affiliations:** Cancer Center, Union Hospital, Tongji Medical College, Huazhong University of Science and Technology, Wuhan, 430022 Hubei China

**Keywords:** Lung adenocarcinoma, Metastasis, Overall survival, Nomogram, Surveillance, Epidemiology and end results

## Abstract

**Objective:**

Metastasis of lung cancer is an important factor affecting survival. The present study proposed to establish and verify a nomogram for predicting overall survival (OS) in lung adenocarcinoma (LUAD) patients with different patterns of metastasis.

**Methods:**

A total of 9727 patients diagnosed with metastatic LUAD patients from 2010 to 2015 were enrolled based on surveillance, epidemiology and end results (SEER) Database and then randomly divided into training and validation cohorts, and 136 patients in our Cancer Center were enrolled as the external validation cohort. Univariate and multivariate analyses were performed to evaluate the prognostic impact on OS. A prognostic nomogram was constructed and evaluated by C-index, calibration curve, decision curve analysis (DCA), and risk stratification system.

**Results:**

Ultimately, 6809 and 2918 patients diagnosed with metastatic LUAD in the training and validation cohorts were enrolled in the study, respectively. A male sex, a later T and N stage, a larger tumor size, treatment including no surgery, no chemotherapy and no radiotherapy, metastasis sites were found to be independent risk factors in LUAD patients for worse OS, and then incorporated into the nomogram. The frequency of bone metastasis was the highest, and in single site metastasis, the prognosis of liver metastasis was the worst. Two-site metastasis is more common than three-site and four-site metastasis, and co-metastasis eventually leads to a worse survival outcome. The C-index value of nomogram for predicting OS were 0.798, 0.703 and 0.698 in the internal training, validation and external validation cohorts, separately. The calibration curves for the 6-months, 1-year and 2-year showed significant agreement between nomogram models and actual observations. The DCA curves indicated nomogram was more beneficial than the AJCC TNM stage. Patients were further divided into low-risk and high-risk groups according to nomogram predicted scores and developed a survival risk classification system.

**Conclusions:**

Our prognostic nomogram is expected to be an accurate and individualized clinical predictive tool for predicting OS in LUAD patients with different patterns of metastasis.

## Introduction

Lung cancer is one of the common malignant tumors that threaten human life, with high morbidity and mortality, accounting for approximately one tenth (11.4%) and one fifth (18.0%) of confirmed cancer and cancer deaths, respectively (Siegel et al. 2022). Non-small cell lung cancer (NSCLC) accounts for about 80% of lung cancer, of which lung adenocarcinoma (LUAD) is the most common subtype (Chen et al. 2014). Early lung cancer can be asymptomatic or atypical, when diagnosed, many patients have progressed to advanced and distant metastasis, which largely determines the treatment strategy and the possibility of long-term survival (Nasim et al. 2019). With the development of gene detection, targeted therapy has made remarkable progress (Jones and Baldwin 2018). In addition, the study of tumor microenvironment has also promoted the progress of immunotherapy to some extent (Hu et al. 2019). Thus, for advanced patients cannot be treated with surgery, the combined use of chemotherapy, radiotherapy, targeted therapy and immunotherapy is recommended to further reduce metastatic risk (Abu Rous et al. 2023). Despite, the prognosis is still not ideal, with their 5-year survival rate is less than 20%, and metastatic lesions are the main reason (Xie et al. 2021).

LUAD is a malignant tumor with major metastatic sites including bone, brain, liver and lung, which has a certain effect on the survival rate (Hendriks et al. 2015). At present, TNM staging is still the gold standard for predicting the prognosis of LUAD, however, patients with the same stage often have different prognosis after receiving similar treatment, probably as some clinical characteristics are not considered in the staging system. Thus, a more accurate prognostic model is needed to provide information for clinical decisions in patients with metastatic LUAD. Recently, as a new statistical prediction model, nomogram has shown good application value in all kinds of cancer (Iasonos et al. 2008), including nasopharyngeal carcinoma (Tang et al. 2018), esophageal cancer (Liu et al. 2021a), colorectal cancer (Liu et al. 2021b), hepatocellular cancer (Liu et al. 2020), small cell lung cancer (Yang et al. 2021) and so on. A previous study (Pang et al. 2022) has constructed a nomogram for predicting distant metastasis in invasive LUAD, however, this study only included whether patients had distant metastasis, no further analysis of specific metastatic sites and different patterns was constructed, and few effective risk stratification tools to optimize the prognostic role of metastasis in LUAD survival were established.

Therefore, in this study, we aim to explore different metastatic patterns of LUAD and their effects on prognosis based on surveillance, epidemiology and end results (SEER) database, and then further evaluate the reliability and feasibility through independent internal cohorts, in order to improve the predictive effectiveness of traditional methods and guide clinical decision-making.

## Materials and methods

### Patients selection

**Fig. 1:**
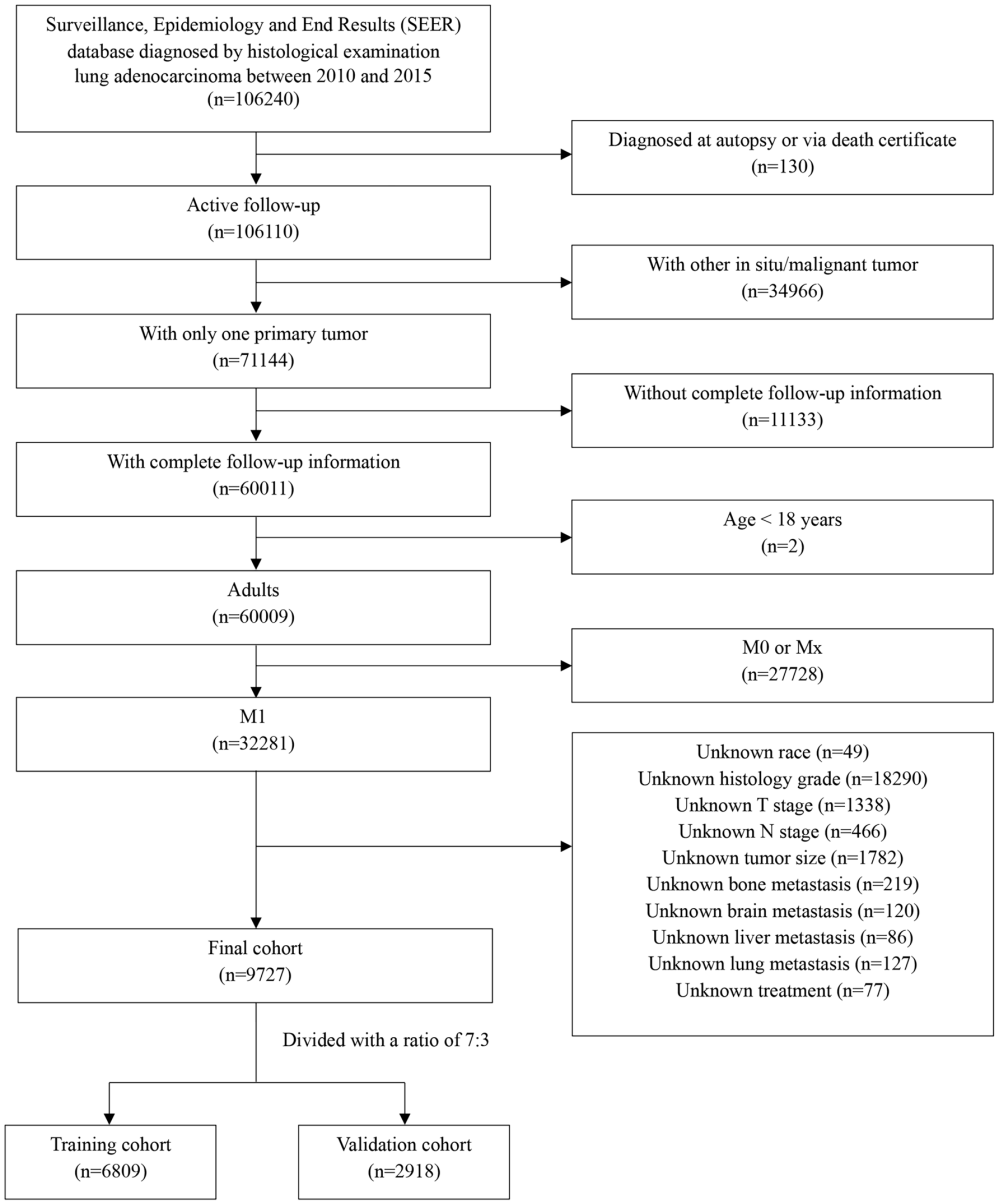
Flow chart of patient selection from the surveillance, epidemiology and end results (SEER) database

This study was conducted as a retrospective study using the SEER database including cancer incidence, survival and treatment information from multiple registries (http://seer.cancer.gov/). The data of patients diagnosed with metastatic LUAD from 2010 to 2015 were enrolled using SEER*Stat version 8.4.1 (username: 25736-Nov2021). Inclusion criteria were the following: (1) patients diagnosed with primary cancer from 2010 to 2015; (2) according to the International Classification of Diseases (ICD) for Oncology-3, patients histological codes were: 8140/3, 8141/3, 8143/3, 8144/3, 8146/3, 8147/3, 8149/3, 8250/3, 8251/3, 8255/3, 8260/3, 8310/3, 8323/3, 8480/3, 8481/3, 8570/3, 8574/3; (3) data such as year of diagnosis, sex, age of diagnosis, race (white, black, other), grade, TNM stage at the time of diagnosis, tumor size, treatment, different patterns of metastatic sites, survival months, and vital survival status were collected from the SEER database. Exclusion criteria included: (1) patients with more than one kind of primary malignant cancer; (2) patients aged < 18 years; (3) patients with survival time less than 1 month; (4) patients with unknown or missing clinical information. The specific inclusion and exclusion processes are shown in Fig. 1. And also, we enrolled patients with metastatic LUAD diagnosed from 2013 to 2016 at Union Hospital Cancer Center as the external validation cohort. Cases without sufficient clinical characteristic information and incomplete follow-up information were excluded. Written consent was obtained from all enrolled patients and the study was approved by Cancer center of Union hospital of Tongji medical college of Huazhong university of science and technology.

### Data collection and clinical endpoints

Demographic and clinicopathological data were extracted in the study: age, sex, race, grade, tumor stage, nodal stage, tumor size, surgery, chemotherapy, radiotherapy, metastatic site and follow-up data. The primary outcome of this study was overall survival (OS), which was defined as the time between first treatment and death or last follow-up. Events that had not occurred by the last follow-up date were recorded as censoring. All patients were followed up by regular records of each clinic recheck or phone calls.

### Statistical analysis

All the analyses were conducted using SPSS 26.0, GraphPad Prism 9.0 and R software v4.2.3. The patients in the training and validation cohorts were divided with a ratio of 7:3 using the “create Data Partition” function in the R “crate” package to ensure that the outcome events were randomly distributed. The Chi-squared test was used to explore the baseline balance between the internal and external cohorts. Correlations between variables were assessed using the Pearson correlation coefficient. Survival curves were analyzed using the Kaplan–Meier method and compared by the log-rank test. Univariate and multivariate Cox proportional hazards regressions were conducted to evaluate the prognostic significance of variables with respect to OS. The nomogram was explored by the “rms” package of R software and the concordance index (C-index) was calculated to predict the performance of the established nomogram, then a calibration curve (1000 bootstrap resampling) to test the calibration and a decision curve analysis (DCA) to evaluate the clinical utility. In addition, the “survminer” package was used to get the cutoff value according to the scores predicted by the nomogram, and then the cohorts were divided into different risk groups to establish the Kaplan–Meier curve. A two-tailed *P* value less than 0.05 was considered statistically significant.

## Results

### Basic patient characteristics in the training and validation cohorts

Ultimately, a total of 9727 patients diagnosed with metastatic LUAD were enrolled in the study, with 6809 and 2918 patients in the training and validation cohorts, respectively. The baseline characteristics of the patients were displayed in Table 1. The training cohort was comprised of 3344 female (49.1%) and 3465 males (50.9%), with the similar proportion in the validation cohort. Besides, metastatic LUAD patients in the two cohorts tend to have a larger tumor size (54.5% in the training and 53.8% in the validation cohort), a later T stage (T3–T4) (63.9% vs 64.1%) and a later N stage (N2–N3) (67.7% vs 67.7%), respectively. At the end of the study period, 681 (10.0%) and 293 (10.0%) patients suffered from death in the two cohorts, separately.

**Table 1:** Baseline characteristics in the SEER training and validation cohorts

Characteristics	Training cohort	Validation cohort	*P* value
	(n = 6809) (%)	(n = 2918) (%)	
Age (years)			0.773
< 65	3452 (50.7)	1490 (51.1)	
65–75	2686 (39.4)	1131 (38.7)	
> 75	671 (9.9)	297 (10.2)	
Sex			0.854
Female	3344 (49.1)	1439 (49.3)	
Male	3465 (50.9)	1479 (50.7)	
Race			0.822
White	5015 (73.7)	2146 (73.5)	
Black	950 (13.9)	419 (14.4)	
Other	844 (12.4)	353 (12.1)	
Grade			0.736
I	499 (7.3)	213 (7.4)	
II	2089 (30.7)	900 (30.8)	
III	4154 (61.0)	1769 (60.6)	
IV	67 (1.0)	36 (1.2)	
Tumor stage			0.996
T1	656 (9.6)	277 (9.5)	
T2	1801 (26.5)	770 (26.4)	
T3	1823 (26.8)	784 (26.9)	
T4	2529 (37.1)	1087 (37.2)	
Nodal stage			0.693
N0	1620 (23.8)	703 (24.1)	
N1	576 (8.5)	240 (8.2)	
N2	3210 (47.1)	1347 (46.2)	
N3	1403 (20.6)	628 (21.5)	
Tumor size			0.832
< 2 cm	594 (8.7)	258 (8.8)	
2–4 cm	2506 (36.8)	1090 (37.4)	
> 4 cm	3709 (54.5)	1570 (53.8)	
Surgery			0.348
Yes	619 (9.1)	248 (8.5)	
No	6190 (90.9)	2670 (91.5)	
Chemotherapy			0.276
Yes	4391 (64.5)	1848 (63.3)	
No	2418 (35.5)	1070 (36.7)	
Radiotherapy			0.543
Yes	3329 (48.9)	1407 (48.2)	
No	3480 (51.1)	1511 (51.8)	
Metastasis			0.910
Unknown	1367 (20.1)	584 (20.0)	
Bone	1083 (15.9)	461 (15.9)	
Brain	1083 (15.9)	474 (16.2)	
Liver	182 (2.7)	77 (2.6)	
Lung	987 (14.5)	468 (16.0)	
Bone and brain	364 (5.3)	156 (5.4)	
Bone and liver	223 (3.3)	85 (2.9)	
Bone and lung	438 (6.4)	187 (6.5)	
Brain and liver	67 (1.1)	23 (0.8)	
Brain and lung	316 (4.6)	131 (4.5)	
Liver and lung	90 (1.3)	35 (1.2)	
Bone, brain and liver	105 (1.5)	40 (1.4)	
Bone, brain and lung	189 (2.8)	82 (2.8)	
Bone, liver and lung	170 (2.5)	60 (2.0)	
Brain, liver and lung	47 (0.7)	16 (0.5)	
Bone, brain, liver and lung	98 (1.4)	39 (1.3)	

In the training cohort, patient with solitary bone, brain, liver and lung metastasis were 1083 (15.9%), 1083 (15.9%), 182 (2.7%) and 987 (14.5%), respectively. Among patient with two metastatic sites, the proportion with both bone and lung metastasis (6.4%) was higher than others. Also, patients with bone, brain and lung metastasis (2.8%) were the most in the three metastatic sites. Besides, the number of LUAD patients with both bone, brain, liver and lung metastasis were 98 (1.4%), which was similar in the validation cohort.

Table 1 showed the correlations between the patient baseline parameters in the two cohorts. As was shown, there was no correlation in age, sex, race, grade, T stage, N stage, tumor size, surgery, chemotherapy, radiotherapy and metastasis, indicating there were no significant differences between the two cohorts. In addition, we enrolled a total of 136 patients with metastatic LUAD at Union Hospital Cancer Center as the external validation cohort. The characteristics between internal and external validation cohorts were compared in Table 2. Similarly, there was no significant difference in the above clinical factors except race (all the patients were Chinese).

**Table 2:** Baseline characteristics in the SEER and external validation cohorts

Characteristics	SEER cohort	External cohort	*P* value
	(n = 9727) (%)	(n = 136) (%)	
Age (years)			0.468
< 65	4942 (50.8)	65 (47.8)	
65–75	3817 (39.2)	60 (44.1)	
> 75	968 (10.0)	11 (8.1)	
Sex			0.509
Female	4783 (49.2)	63 (46.3)	
Male	4944 (50.8)	73 (53.7)	
Race			< 0.001*
White	7161 (73.6)	0 (0.0)	
Black	1369 (14.1)	0 (0.0)	
Other	1197 (12.3)	136 (100.0)	
Grade			0.670
I	712 (7.3)	9 (6.6)	
II	2989 (30.7)	36 (26.5)	
III	5923 (60.9)	89 (65.4)	
IV	103 (1.1)	2 (1.5)	
Tumor stage			0.132
T1	933 (9.6)	6 (4.4)	
T2	2571 (26.4)	44 (32.4)	
T3	2607 (26.8)	35 (25.7)	
T4	3616 (37.2)	51 (37.5)	
Nodal stage			0.635
N0	2323 (23.9)	30 (22.1)	
N1	816 (8.3)	9 (6.6)	
N2	4557 (46.8)	71 (52.2)	
N3	2031 (21.0)	26 (19.1)	
Tumor size			0.858
< 2 cm	852 (8.8)	11 (8.1)	
2–4 cm	3596 (37.0)	48 (35.3)	
> 4 cm	5279 (54.2)	77 (56.6)	
Surgery			0.737
Yes	867 (8.9)	11 (8.1)	
No	8860 (91.1)	125 (91.9)	
Chemotherapy			0.229
Yes	6239 (64.1)	94 (69.1)	
No	3488 (35.9)	42 (30.9)	
Radiotherapy			0.244
Yes	4736 (48.7)	73 (53.7)	
No	4991 (51.3)	63 (46.3)	
Metastasis			0.119
Unknown/others	1951 (20.1)	27 (19.9)	
Bone	1544 (15.9)	13 (9.6)	
Brain	1557 (16.0)	26 (19.1)	
Liver	259 (2.7)	1 (0.7)	
Lung	1455 (14.9)	22 (16.2)	
Bone and brain	520 (5.3)	5 (3.7)	
Bone and liver	308 (3.2)	6 (4.4)	
Bone and lung	625 (6.4)	3 (2.2)	
Brain and liver	90 (0.9)	3 (2.2)	
Brain and lung	447 (4.6)	8 (5.9)	
Liver and lung	125 (1.3)	1 (0.7)	
Bone, brain and liver	145 (1.5)	2 (1.5)	
Bone, brain and lung	271 (2.8)	11 (8.1)	
Bone, liver and lung	230 (2.4)	4 (2.9)	
Brain, liver and lung	63 (0.6)	1 (0.7)	
Bone, brain, liver and lung	137 (1.4)	3 (2.2)	

*Statistically significant

### Kaplan–Meier method and log-rank test

**Fig. 2:**
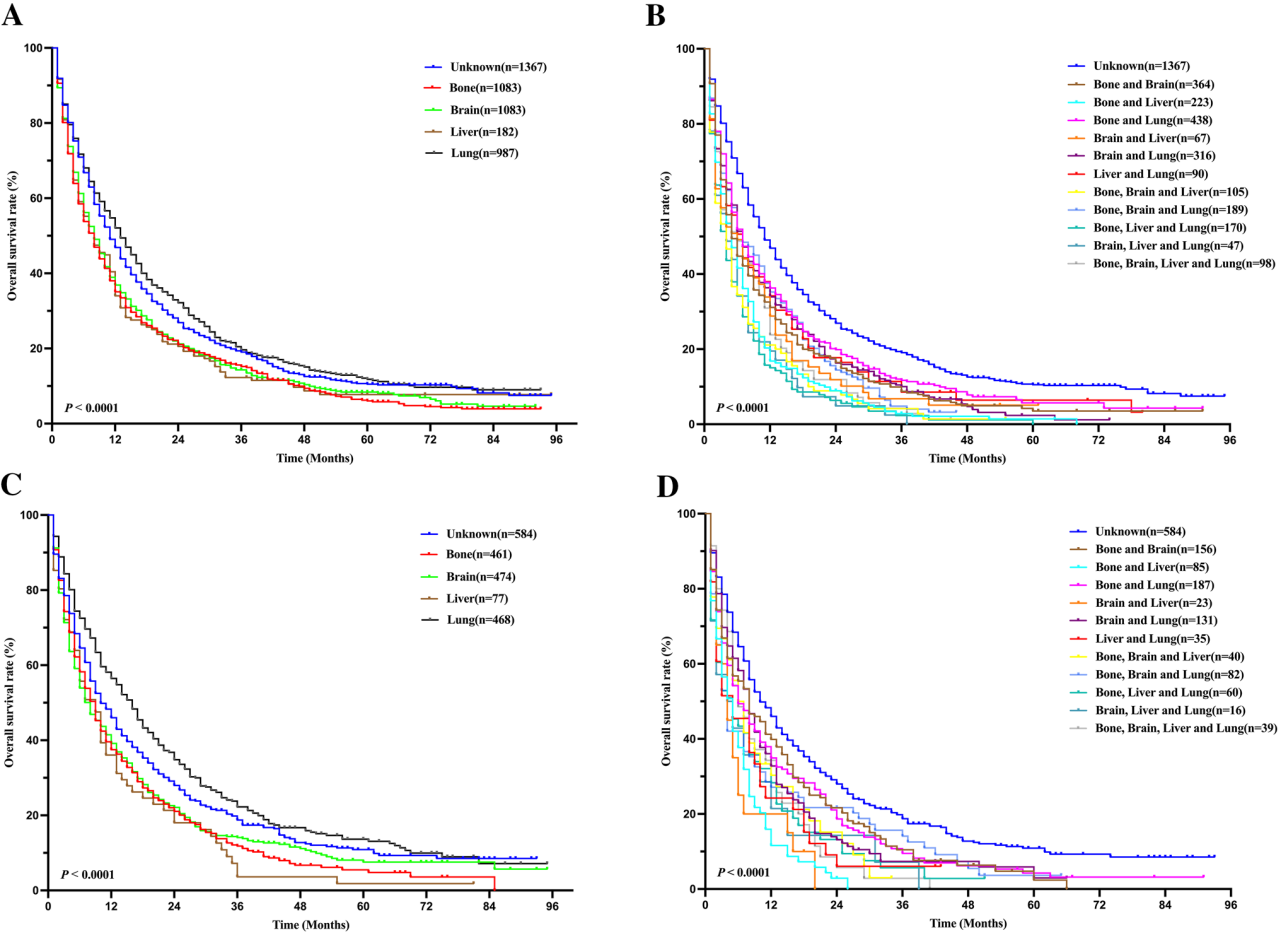
Kaplan–Meier survival curves of overall survival according to single site (**a**, **c**) and multiple site metastasis (**b**, **d**) in the training and validation cohorts. Log-rank test, *P* < 0.05 was considered statistically significant

**Fig. 3:**
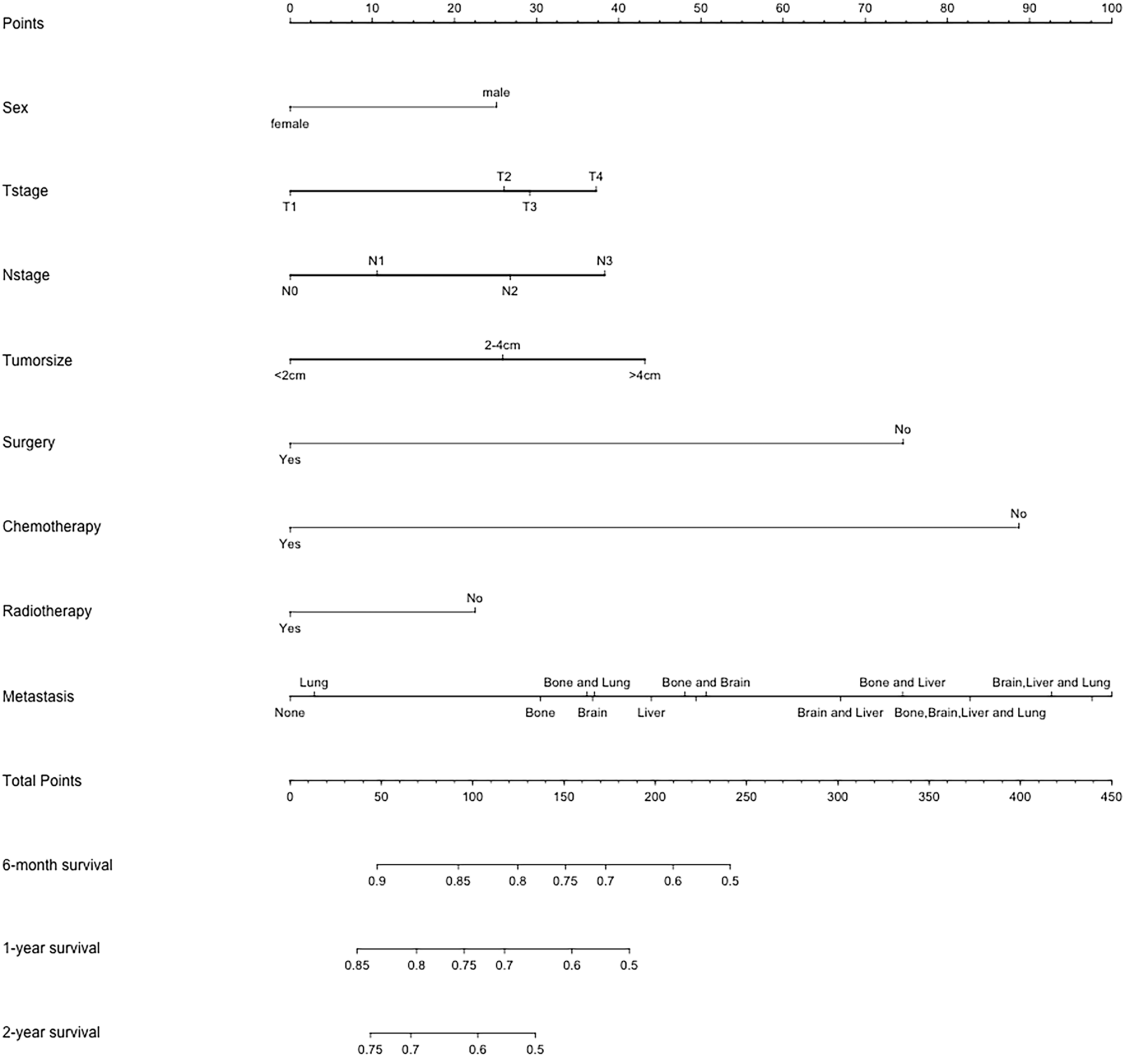
Nomogram for predicting 6-month, 1-year, and 2-year overall survival in LUAD patients with metastasis in the training cohort. The metastatic site on the nomogram from left to right is: unknown; lung; bone; bone and lung; brain; brain and lung; liver; bone, brain and lung; liver and lung; bone and brain; brain and liver; bone and liver; bone, brain, liver and lung; brain, liver and lung; bone, liver and lung; bone, brain and liver

To further investigate the prognosis of metastasis in LUAD patients, survival curves based on OS and different metastatic sites were analyzed in the training and validation cohorts using the Kaplan–Meier method and compared by the log-rank test (Fig. 2). In the training cohort, we found that the frequency of bone metastasis (39.1%) was the highest, and in solitary site metastasis, the prognosis of liver metastasis was the worst (*P* < 0.001, Fig. 2a, c). Two-site metastasis (22%) is more common than three-site (7.5%) and four-site metastasis (1.4%). And in the cases with multiple metastatic sites, the clinical outcomes of the cases with bone, brain and liver metastasis were the worst (*P* < 0.001, Fig. 2b, d), eventually inferior to the single sites. These results were nearly similar in the validation cohort (Fig. 3).

### Univariate and multivariate analysis

In the univariate Cox regression analysis, age, sex, T stage, N stage, tumor size, treatment including surgery, chemotherapy and radiotherapy, metastasis sites besides single lung were corroborated as potential factors affecting OS in the training and validation cohorts (Tables 3, 4). Parameters that reached a significant difference in the univariate analysis were further analyzed considering the influence of confounding factors. As demonstrated in the table, the above variables except age are included after analysis in the multivariate Cox regression analysis. Fortunately, a male sex, a later T and N stage, a larger tumor size, treatment including no surgery, no chemotherapy and no radiotherapy, metastasis sites were still found to be independent risk factors in LUAD patients for worse OS in the two cohorts (All *P* values < 0.05).

**Table 3:** Univariate and multivariate cox analyses on variables for the prediction of overall survival in the training cohort

Characteristics	Univariate analysis		Multivariate analysis	
	HR (95% CI)	*P*	HR (95% CI)	P
Age (years)				
< 65	Reference			
65–75	1.214(1.151–1.280)	< 0.001*		0.075
> 75	1.373(1.260–1.497)	< 0.001*		
Sex				
Female	Reference		Reference	
Male	1.292(1.229–1.359)	< 0.001*	1.254(1.192–1.319)	< 0.001*
Race				
White	Reference			
Black	1.073(0.998–1.153)	0.056		0.194
Other	0.673(0.621–0.729)	< 0.001*		
Grade				
I	Reference			
II	1.106(0.995–1.229)	0.063		0.753
III	1.495(1.352–1.653)	< 0.001*		
IV	1.639(1.258–2.136)	< 0.001*		
Tumor stage				
T1	Reference		Reference	
T2	1.191(1.083–1.310)	< 0.001*	1.247(1.132–1.372)	< 0.001*
T3	1.226(1.115–1.349)	< 0.001*	1.289(1.169–1.421)	< 0.001*
T4	1.328(1.212–1.455)	< 0.001*	1.374(1.248–1.512)	< 0.001*
Nodal stage				
N0	Reference		Reference	
N1	1.116(1.008–1.235)	0.035*	1.072(0.967–1.188)	0.047*
N2	1.323(1.241–1.411)	< 0.001*	1.245(1,164–1.331)	< 0.001*
N3	1.429(1.324–1.542)	< 0.001*	1.366(1.261–1.479)	< 0.001*
Tumor size				
< 2 cm	Reference		Reference	
2–4 cm	1.352(1.225–1.493)	< 0.001*	1.250(1.131–1.380)	< 0.001*
> 4 cm	1.578(1.433–1.737)	< 0.001*	1.326(1.153–1.525)	< 0.001*
Surgery				
No	Reference		Reference	
Yes	0.469(0.426–0.517)	< 0.001*	0.500(0.452–0.553)	< 0.001*
Chemotherapy				
No	Reference		Reference	
Yes	0.428(0.406–0.451)	< 0.001*	0.443(0.402–0.488)	< 0.001*
Radiotherapy				
No	Reference		Reference	
Yes	0.641(0.610–0.674)	< 0.001*	0.835(0.751–0.927)	0.001*
Metastasis				
Unknown	Reference		Reference	
Bone	1.220(1.121–1.328)	< 0.001*	1.334(1.211–1.470)	< 0.001*
Brain	1.154(1.060–1.256)	0.001*	1.375(1.235–1.532)	< 0.001*
Liver	1.307(1.111–1.537)	0.001*	1.419(1.191–1.691)	< 0.001*
Lung	0.942(0.863–1.029)	0.018*	1.048(0.938–1.170)	0.406
Bone and brain	1.418(1.257–1.600)	< 0.001*	1.655(1.441–1.900)	< 0.001*
Bone and liver	1.879(1.626–2.171)	< 0.001*	1.965(1.676–2.304)	< 0.001*
Bone and lung	1.290(1.151–1.445)	< 0.001*	1.420(1.243–1.622)	< 0.001*
Brain and liver	1.643(1.278–2.113)	< 0.001*	1.771(1.365–2.298)	< 0.001*
Brain and lung	1.392(1.226–1.581)	< 0.001*	1.428(1.237–1.649)	< 0.001*
Liver and lung	1.445(1.159–1.800)	0.001*	1.626(1.290–2.049)	< 0.001*
Bone, brain and liver	2.096(1.713–2.564)	< 0.001*	2.491(2.012–3.083)	< 0.001*
Bone, brain and lung	1.419(1.212–1.661)	< 0.001*	1.681(1.413–1.999)	< 0.001*
Bone, liver and lung	2.253(1.912–2.654)	< 0.001*	2.461(2.059–2.940)	< 0.001*
Brain, liver and lung	2.156(1.611–2.887)	< 0.001*	2.391(1.770–3.230)	< 0.001*
Bone, brain, liver and lung	1.804(1.465–2.222)	< 0.001*	2.173(1.740–2.713)	< 0.001*

*Statistically significant

### Construction of nomogram

To further analyze the prognostic values of risk factors, we created the nomogram model that incorporated all significant factors in the multivariate Cox regression analysis (Fig. 3). As shown in the nomogram, a male gender, patients with T4 or N3 stage, tumor larger than 4 cm, no surgery, no chemotherapy or radiotherapy, patients with bone, brain and liver metastasis were demonstrated as the favorable parameters to prognosis. And the 6-month, 1-year and 2-year OS was predicted in this nomogram based on chosen variables that had a hazard ratio. All the prediction parameters have corresponding accurate values in the nomogram. Add all these values and put them in the total score scale to calculate the survival probability.

### Calibration and validation of the nomogram

**Fig. 4:**
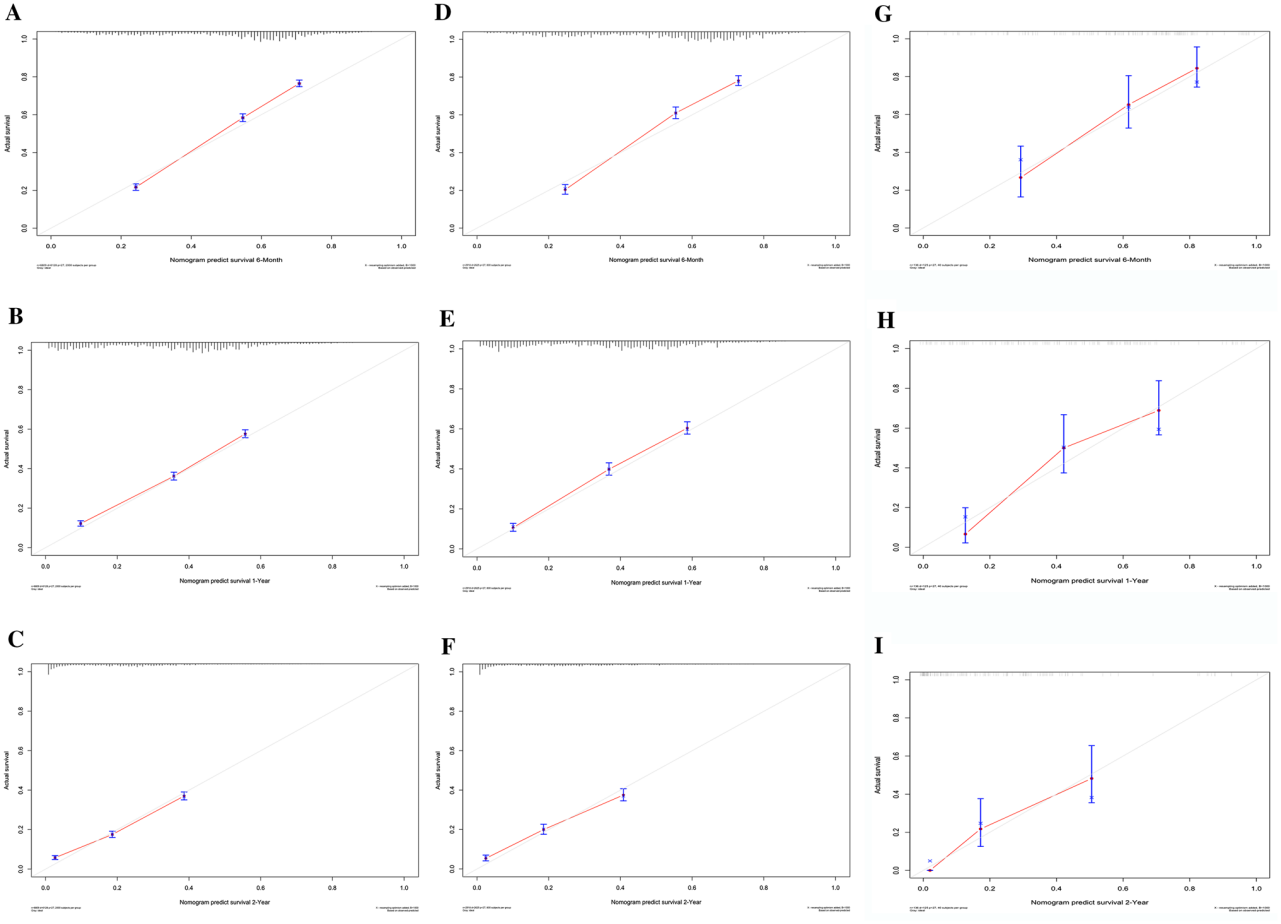
Calibration curves for the nomogram in the training cohort (**a**–**c**), internal validation (**d**–**f**) and external validation (**g**–**i**) cohorts for 6-month, 1-year, and 2-year

Generally, the C-index is used to quantify the prediction ability of the nomogram model, and the values were 0.798, 0.703 and 0.698 in the internal training, validation and external validation cohorts, separately. And then, the nomogram calibration curves were constructed for 6-month, 1-year and 2-year in the cohorts, respectively, which demonstrated a high degree of consistency between the anticipated and actually observed survival probabilities (Fig. 4).

**Fig. 5:**
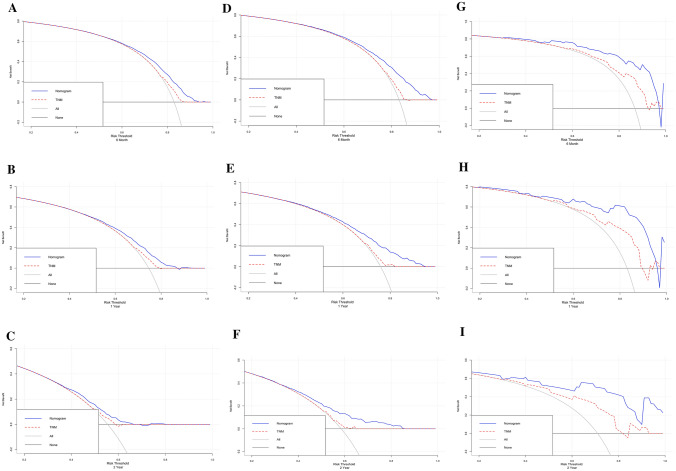
DCA curves for the nomogram in the training cohort (a–**c**), internal validation (**d**–**f**) and external validation (**g**–**i**) cohorts for 6-month, 1-year, and 2-year

Normally, the DCA curve was developed to identify the clinical benefits and utility of the nomogram compared to the TNM staging system. As well, the DCA curves of the cohorts both demonstrated that the nomogram we constructed in predicting OS was more beneficial than the 7th American Joint Committee on Cancer (AJCC) TNM stage, and also displaying net benefit in predictive models for threshold probabilities at different time points (Fig. 5). To sum up, the above results showed that the nomogram we constructed had pronounced discriminative and calibration capabilities in metastatic LUAD patients.

### Risk stratification of prognosis by the nomogram model

**Fig. 6:**
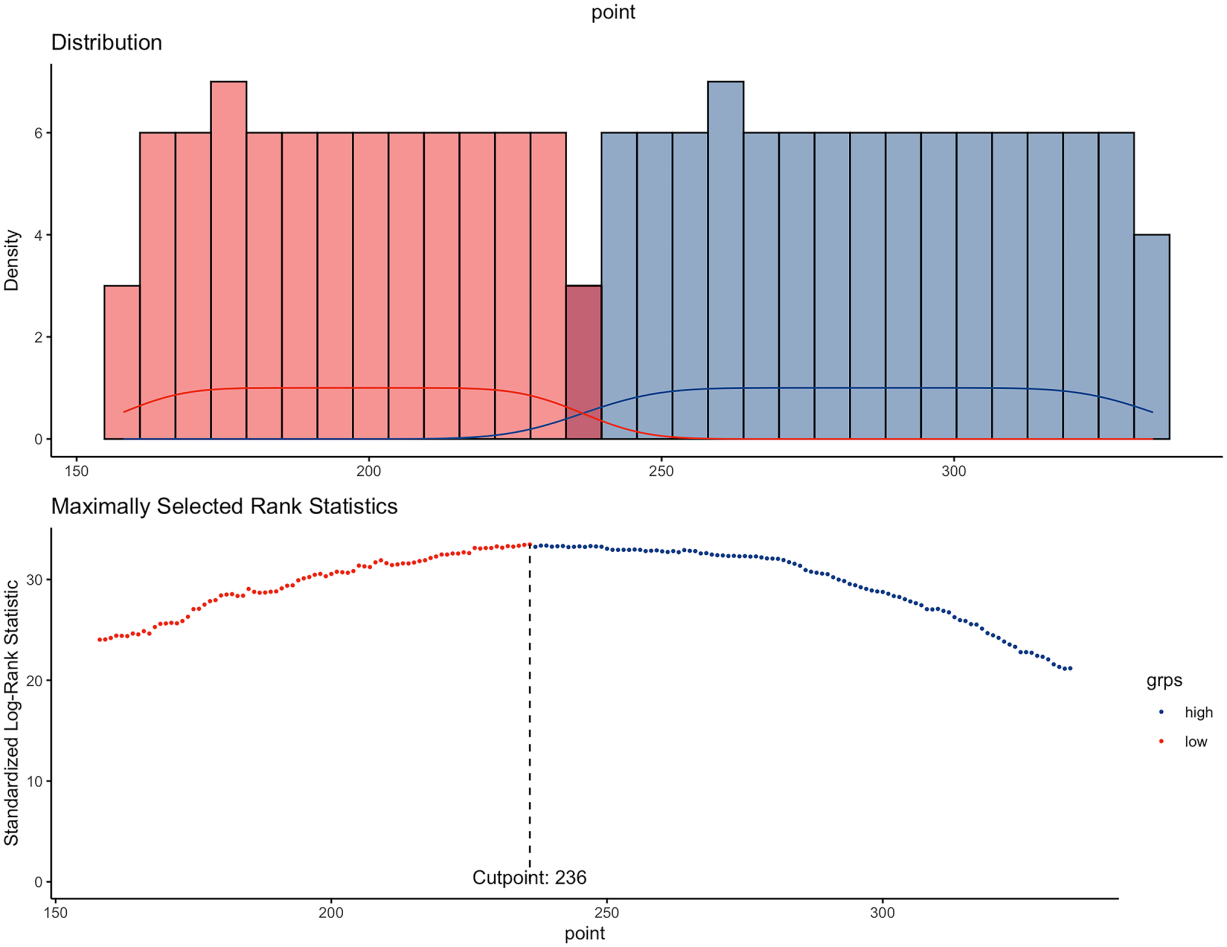
Risk stratification system based on nomogram predicted scores and cut off value by R software in the training cohort

**Fig. 7:**
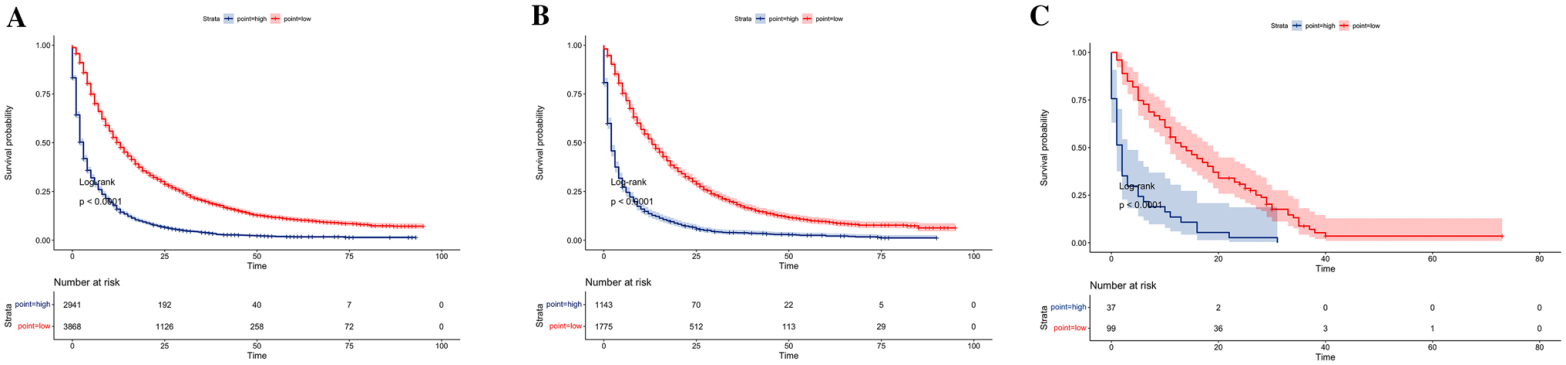
Kaplan–Meier survival curves categorized into low-risk (total risk score < 236) and high-risk groups (total risk score ≥ 236) based on cut off value according to prognostic score of the nomogram in the training (**a**), internal validation (**b**) and external validation (**c**) cohorts. Log-rank test, P < 0.05 was considered statistically significant

Finally, a risk stratification system for predicting OS based on the total nomogram scores was developed. First, the total risk score for each case was calculated in the training cohort, and then the cut-off value was 236 according to R software (Fig. 6). Based on the cut-off value, included patients were divided into low and high-risk groups. Further analyze in the internal and external cohorts were demonstrated that, for metastatic LUAD patients, those in the low-risk group (total risk score < 236) had superior prognosis than the high-risk group (total risk score ≥ 236) (*P* < 0.0001) (Fig. 7).

## Discussion

Metastasis is the main cause of lung cancer-related death (Nichols et al. 2021), with 40–60% of lung cancer patients already developing metastasis when diagnosed, leading to a poor prognosis (Stein et al. 2021). At present, the gold standard used to predict the prognosis of lung cancer is the TNM staging system, and all lung cancer patients with metastasis are classified as stage IV, whose first-line treatment is still a combination of chemotherapy, radiotherapy, immunotherapy and targeted therapy (Gadgeel et al. 2012). Unfortunately, patients with the same stage often have a heterogeneous prognosis, as some potential prognostic factors are not considered in the TNM staging system. And previous studies have shown that there are great differences in tumor characteristics, metastasis patterns and prognosis among different histological types of lung cancer (Wang et al. 2020). NSCLC accounts for about 80% of lung cancer, of which LUAD is the most common subtype, with a 5-year survival rate less than 20% (Torre et al. 2016). Therefore, early identification of high-risk metastatic LUAD patients has important guiding significance for treatment decision-making, long-term survival assessment and follow-up frequency.

At present, the nomogram has been considered as a useful tool to assess risk by integrating the important pathological and clinical features of oncology results (Zuo et al. 2021). Some studies (Ouyang et al. 2022) have constructed nomograms to predict the survival prognosis of patients with lung cancer by integrating different clinical factors, and have shown good reliability and feasibility. In addition, for metastatic LUAD patients, Pang et al. (2022) established a nomogram by incorporating factors such as histological type, surgical approach and metastatic status to accurately predict prognosis. However, this study only included whether patients had distant metastasis, no further analysis of specific metastatic sites and different patterns was constructed. Go a step further, single metastatic site was enrolled to construct different nomogram for LUAD patients in several studies. Meng et al. (2022) investigated pretreatment peripheral blood indexes in advanced LUAD with bone-only metastasis and developed a nomogram model to estimate survival. A nomogram for predicting brain metastasis of EGFR-mutated LUAD patients and estimating the efficacy of therapeutic strategies was constructed by Wang et al. (2021) Similarly, a nomogram model was designed by Wang et al. (2022a) based on easily accessible clinical factors which demonstrated excellent performance to predict the individual cancer-specific survival of NSCLC patients with liver metastasis. According to the above studies, we found that tumor metastasis has been considered to be an important prognostic factor in LUAD patients, but its impact in patients with multiple sites has not been comprehensively analyzed and predicted. Thus, we aim to explore different metastasis patterns of LUAD and their effects on survival prognosis.

In this study, we demonstrated that clinical parameters including sex, T stage, N stage, tumor size, treatment including surgery, chemotherapy and radiotherapy had a significant impact on patient survival, which was consistent with previous studies (Deng et al. 2018; Wang et al. 2022b). Whereas, age is not an independent factor affecting distant metastasis, which may be related to the differences of subjects. At the same time, we found that LUAD most frequently metastasized to bone (39.1%), lung (34.2%), brain (33.6%) and liver (14.5%), and also, in solitary metastatic site, patients with single liver metastasis demonstrated poorer OS, similar to the former research (Wang et al. 2023). To our knowledge, for patients with liver metastasis, chemotherapy and radiotherapy are the standard treatment, however, the inability to tolerate chemotherapy due to liver insufficiency caused by liver metastasis may also lead to a poor prognosis (Nakagawa et al. 2008). At the same time, this also may be explained by the liver is an immunosuppressive organ, which hinders the immune surveillance of other metastatic organs when liver metastasis occurs (Ham et al. 2015). Therefore, whenever a patient has liver metastasis, no matter where it is combined, there is a tendency to have a poor prognosis compared with other modes without liver metastasis, fortunately, which has also been confirmed in our study. Nevertheless, a significant difference in patients with single lung metastasis was not found in the nomogram. Interestingly, in the subgroup analysis of evaluating the risk of major organ metastasis of different histological types of lung cancer by Wang et al. (2023), it was found that small-cell lung carcinoma (SCLC), large-cell carcinoma (LCLC), squamous-cell carcinoma (SCC) could increase or decrease the risk of lung metastasis to varying degrees, but there was no correlation in LUAD, which may be related to our trouble to get the expected results. What is more, apart from single site, we further integrated multi-site metastasis into the nomogram. As we have studied, two-site metastasis is more common than three-site and four-site metastasis, cases with bone, brain and liver metastasis were found to obtain the worst survival prognosis, and co-metastasis eventually leads to a worse survival outcome compared to single sites.

At the same time, the nomogram in our study also showed better predictive accuracy and differential ability to predict the survival rate of metastatic LUAD patients. Further, DCA curves also confirmed that the nomogram we constructed was more beneficial than the 7th AJCC TNM stage in predicting 6-month, 1-and 2-year OS. Finally, patients were divided into two risk groups according to the total score based on the nomogram, and significant survival differences were found in Kaplan–Meier curve evaluation.

Nevertheless, our study also has some limitations. First, there is a lack of some important clinicopathological factors in the SEER database, such as specific treatment regimens, tumor markers, gene mutations and so on. Secondly, as a retrospective study, selection bias is inevitable. In addition, although we have fully considered metastatic sites, there is a lack of information on the number of metastatic lesions, so we are unable to incorporate this important factor into the model. Therefore, larger sample size, multicenter clinical studies are needed to further confirm the model (Table 4).

**Table 4:** Univariate and multivariate cox analyses on variables for the prediction of overall survival in the validation cohort

Characteristics	Univariate analysis		Multivariate analysis	
	HR (95% CI)	*P*	HR (95% CI)	*P*
Age (years)				
< 65	Reference			
65–75	1.268(1.169–1.376)	< 0.001*		0.722
> 75	1.276(1.120–1.454)	< 0.001*		0.208
Sex				
Female	Reference		Reference	
Male	1.279(1.184–1.381)	< 0.001*	1.224(1.133–1.324)	< 0.001*
Race				
White	Reference			
Black	1.101(0.987–1.229)	0.085		0.382
Other	0.631(0.557–0.715)	< 0.001*		
Grade				
I	Reference			
II	1.212(1.030–1.426)	0.021*		0.082
III	1.693(1.450–1.978)	< 0.001*		0.431
IV	1.525(1.051–2.211)	0.026*		0.392
Tumor stage				
T1	Reference		Reference	
T2	1.222(1.054–1.417)	0.008*	1.203(1.036–1.396)	0.015*
T3	1.301(1.122–1.507)	< 0.001*	1.290(1.109–1.501)	0.001*
T4	1.316(1.141–1.517)	< 0.001*	1.293(1.114–1.501)	0.001*
Nodal stage				
N0	Reference		Reference	
N1	1.252(1.071–1.463)	0.005*	1.297(1.107–1.519)	0.001*
N2	1.382(1.253–1.525)	< 0.001*	1.398(1.264–1.547)	< 0.001*
N3	1.434(1.278–1.608)	< 0.001*	1.493(1.324–1.683)	< 0.001*
Tumor size				
< 2 cm	Reference		Reference	
2–4 cm	1.316(1.135–1.527)	< 0.001*	1.233(1.061–1.432)	0.006*
> 4 cm	1.577(1.365–1.821)	< 0.001*	1.497(1.291–1.736)	< 0.001*
Surgery				
No	Reference		Reference	
Yes	0.466(0.399–0.544)	< 0.001*	0.521(0.444–0.611)	< 0.001*
Chemotherapy				
No	Reference		Reference	
Yes	0.400(0.369–0.433)	< 0.001*	0.457(0.403–0.519)	< 0.001*
Radiotherapy				
No	Reference		Reference	
Yes	0.549(0.508–0.594)	< 0.001*	0.743(0.654–0.844)	0.001*
Metastasis				
Unknown	Reference		Reference	
Bone	1.174(1.032–1.337)	0.015*	1.029(0.898–1.180)	0.038*
Brain	1.128(0.991–1.284)	0.028*	1.057(0.924–1.209)	0.047*
Liver	1.510(1.184–1.924)	0.001*	1.251(0.974–1.608)	0.008*
Lung	0.853(0.748–0.924)	0.069	0.819(0.715–0.938)	0.104
Bone and brain	1.226(1.019–1.475)	0.031*	1.256(1.042–1.514)	0.017*
Bone and liver	2.361(1.871–2.980)	< 0.001*	2.221(1.756–2.808)	< 0.001*
Bone and lung	1.329(1.120–1.578)	0.001*	1.262(1.059–1.504)	0.009*
Brain and liver	2.202(1.436–3.378)	< 0.001*	2.039(1.325–3.136)	0.001*
Brain and lung	1.350(1.108–1.645)	0.003*	1.456(1.191–1.779)	< 0.001*
Liver and lung	1.609(1.131–2.289)	0.008*	1.492(1.045–2.128)	0.027*
Bone, brain and liver	1.602(1.151–2.229)	0.005*	1.564(1.119–2.185)	0.009*
Bone, brain and lung	1.437(1.129–1.829)	0.003*	1.556(1.217–1.987)	< 0.001*
Bone, liver and lung	1.740(1.325–2.284)	< 0.001*	1.731(1.314–2.281)	< 0.001*
Brain, liver and lung	1.830(1.112–3.012)	0.017*	1.566(0.949–2.583)	0.039*
Bone, brain, liver and lung	1.631(1.177–2.260)	0.003*	1.783(1.282–2.479)	0.001*

*Statistically significant

## Conclusion

In summary, based on the SEER database, we successfully constructed a nomogram including different metastatic patterns to predict the OS of LUAD patients. We found that the frequency of bone metastasis was the highest, and in single site, the prognosis of liver metastasis was the worst. Two-site metastasis is more common than three-site and four-site metastasis, and co-metastasis eventually leads to a worse survival outcome. What is more, risk models and nomogram are more accurate than the TNM staging system in predicting OS for LUAD patients with metastasis. And also, the risk stratification system based on the nomogram is a useful tool to guide metastatic LUAD decision-making and predict clinical outcomes.

## Data Availability

The datasets generated during the current study are available from the corresponding author on reasonable request.
